# Dysmenorrhea due to undiagnosed obstructed hemi-vagina and ipsilateral renal anomaly syndrome can become a cause of suicide

**DOI:** 10.1265/ehpm.21-00043

**Published:** 2022-03-04

**Authors:** Akari Takaya Uno, Ken-ichi Mukaisho, Masahito Hitosugi

**Affiliations:** 1 Department of Legal Medicine, Shiga University of Medical Science; 2 Department of Pathology, Shiga University of Medical Science

**Keywords:** Adolescent women, Dysmenorrhea, Forensic pathology, Obstructed hemi-vagina and ipsilateral renal anomaly (OHVIRA) syndrome, Suicide

## Abstract

**Background:**

A Japanese woman in her early twenties had committed suicide, jumped from a 25-meter high bridge into a lake. She had been suffering from severe dysmenorrhea and general fatigue monthly.

**Results:**

A forensic autopsy revealed indications of a bicorporeal uterus, obstructed hemi-vagina, and ipsilateral renal agenesis, which lead to a diagnosis of obstructed hemi-vagina and ipsilateral renal anomaly (OHVIRA) syndrome. On the right side of the uterus, an enclosed cavity composed of black clots was observed. Histological findings suggested that her endometrium was in the early proliferative phase, implying that she was in the menstrual phase just before her death. She may have been suffering from severe lower abdominal pain from the increased pressure of the closed uterus cavity.

**Conclusions:**

This case indicates that dysmenorrhea from undiagnosed OHVIRA syndrome can possibly lead to a suicide attempt. In Japan, because suicide was the leading cause of death for people aged 15 to 39 in 2019, preventive measures for suicide should be promoted. The present case also suggests that intervention for dysmenorrhea may prevent this in adolescent woman.

**Dear Editor,**

In Japan, suicide was the leading cause of death for people aged 15 to 39 in 2019. Although the number of suicides decreased from 29,554 in 2010 to 19,415 in 2019, it has increased to 21,081 in 2020 likely from the coronavirus disease 2019 (COVID-19) pandemic [1]. The suicide rate in Japan (23.6 per 100,000 in men and 9.6 per 100,000 in women) revealed higher values than other developed countries [1]. Therefore, preventive measures for suicide should be promoted in Japan.

Here, we describe an autopsy case of a young woman who committed suicide after suffering from severe dysmenorrhea before death. Owing to the present results, we emphasize that intervention for severe dysmenorrhea should be part of preventive measures for suicide in adolescent women.

A Japanese woman, a college student in her early twenties, jumped from a 25-meter high bridge into a lake. She was found dead and her body was recovered from the lake. According to her family, she had been suffering from severe dysmenorrhea and general fatigue monthly, but had not been referred to a physician. No other symptoms were observed and she had never undergone systematic radiologic examinations. She also had no history of gravidity. No other causes of suicide attempt were found according to the in-depth police investigation. To determine the detailed cause of death, a forensic autopsy was performed on her body the following day.

At autopsy, a right 2^nd^ rib fracture, bilateral lung contusions, and liver subcapsular and mesenteric hemorrhages, consistent with impacting the surface of the lake, were found. In the trachea and bronchi, an amount of reddish-brown fluid was found. The lungs were enlarged and completely occupied their respective pleural cavities. The left and right lungs weighed 474.8 g and 496.5 g, respectively. Edematous changes in the hilar regions and emphysema characteristics in the peripheral regions were also observed.

The right kidney was absent and the left kidney showed compensatory enlargement with a weight of 213.3 g. A complete bicorporeal uterus (previously called didelphys uterus) with a size of 7.0 × 3.3 × 1.9 cm on the left and 5.0 × 2.5 × 2.0 cm on the right was observed (Fig. 1-A). The left side of the uterus cavity was in communication with the cervix and vagina. However, the right side of the uterus displayed an enclosed cavity that did not communicate with the cervix. Black clots were involved in both uterus cavities (Fig. 1-B). Histologically, an endometrium with proliferative glands was observed in both sides of the uterus. The endometrium was composed of straight, relatively uncoiled glands, and the epithelium was pseudostratified. These histological features suggested the early proliferative phase (Fig. 2-A). Hemorrhages in the endometrium were observed in both the right and left sides of the uterus. Hemorrhages on the surface of the myometrium with accumulation of hemosiderin were also observed in the right side of the uterus (Fig. 2-B). No abnormalities were seen in the ovaries.

**Fig. 1:**
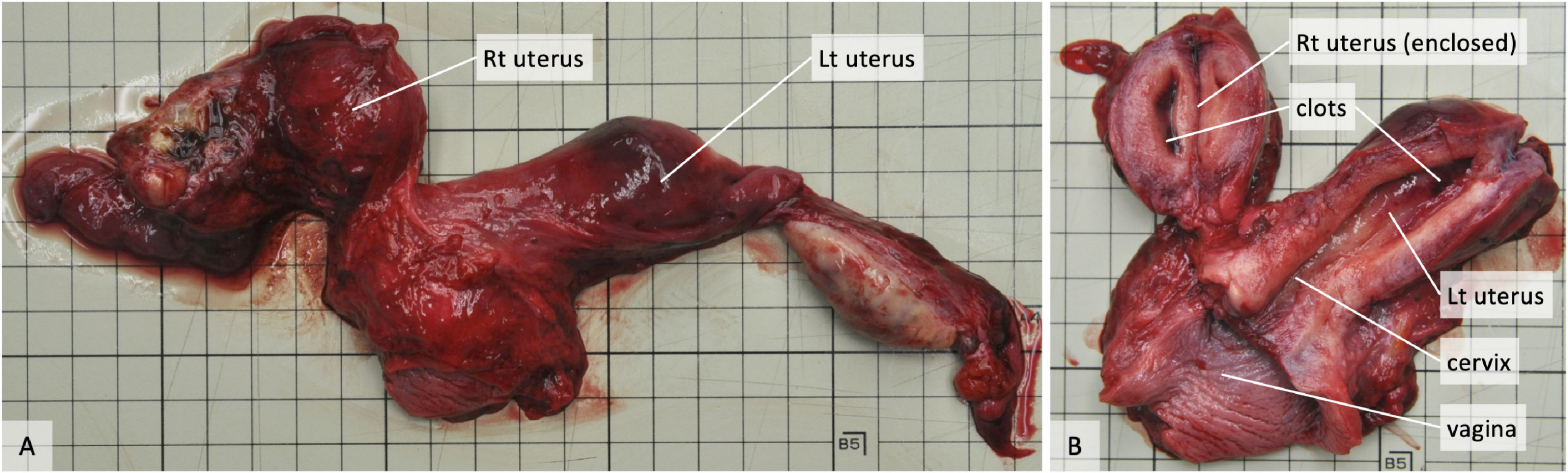
Macroscopic images of the uterus, ovaries and salpinx. Macroscopic findings of the bicorporeal uterus, normal ovaries, and normal salpinx (A). The left side of the uterus communicated with the normal cervix and normal vagina, while the right side of the uterus was enclosed (B).

**Fig. 2:**
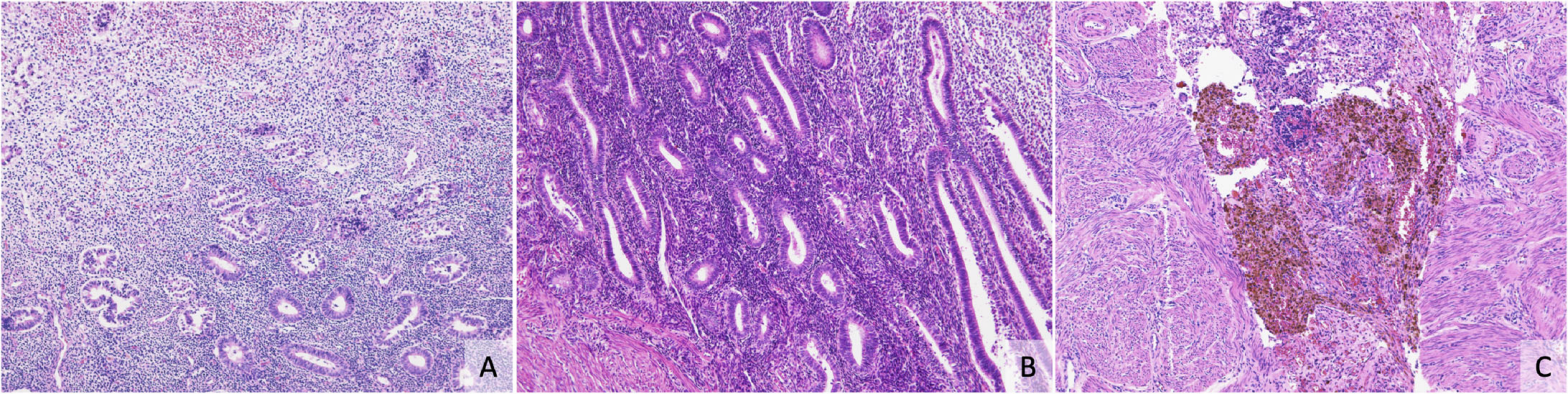
Light microscopic views of the endometrium in the bicorporeal uterus. Histological findings of the endometrium in the left side of the uterus (A) (100 X magnification) and right side of the uterus (B). The right side of the uterus revealed hemorrhages on the surface of the myometrium with accumulation of hemosiderin with hematoxylin and eosin staining (100X magnification).

Diatom studies showed the presence of numerous diatoms in the lungs, left kidney, liver, spleen, and stomach contents. Toxicologically, neither alcohol nor other illicit drugs were found in the blood or urine.

From the autopsy and toxicologic, histological, and diatom examinations, the woman’s cause of death was determined to be drowning. Furthermore, on the basis of the autopsy findings with the bicorporeal uterus, obstructed hemi-vagina, and ipsilateral renal agenesis, the woman was diagnosed with OHVIRA syndrome. Additionally, the histological findings of hemorrhages in the endometrium, particularly in the right side of the uterus (closed cavity), were compatible with the woman suffering from dysmenorrhea.

A variety of factors, including economic problems, unemployment, violence, and health problems, are known to contribute to the suicide rate [2, 3]. Dysmenorrhea is the most common gynecological complaint and has a major impact on quality of life [4]. Previously, dysmenorrhea has been strongly linked with depression among adolescents [5, 6]. According to a retrospective analysis of female suicides in India, abdominal pain was the most common reason for committing suicide [7]. Another case control study suggested that dysmenorrhea was the leading cause of suicide attempts from physical disorders [8]. Therefore, dysmenorrhea can become a trigger of suicide in adolescent women.

OHVIRA syndrome is a rare clinical entity of Müllerian anomalies, estimated to affect approximately 0.1%–3.8% of females in the United States [9]. It mainly presents with cyclical and/or chronic pelvic pain and pelvic swelling during regular cycles caused by hematometrocolpos in the obstructed hemi-vagina [10].

Although lower abdominal pain frequently occurs during each menstrual cycle, the diagnosis is often delayed without referring gynecologists because the menstrual cycles are regular. The symptoms are considered to be typical dysmenorrhea.

In this case, according to the police investigation, the victim had been complaining of severe dysmenorrhea and general fatigue, but no warning was found prior to death. At autopsy, OHVIRA syndrome was confirmed and the right side of the uterus was not in communication with the cervix. Histological findings revealing that her endometrium was in the early proliferative phase suggested that she had been in the menstrual phase just before her death. The woman may have been suffering from severe lower abdominal pain as a result of the increased pressure of the closed uterus cavity. Therefore, we now indicate that undiagnosed OHVIRA syndrome can become a cause of suicide attempt in adolescent women.

Because there are a variety of anomalies frequently associated with OHVIRA syndrome, some patients have no marked symptoms or complaints. However, in this case, endometrium tissues were observed in the closed uterus cavity, so the victim was believed to have had numerous complaints because of hemorrhages during the menstrual phase. Perhaps if she had visited a gynecologist and received adequate intervention, the suicide attempt could possibly have been avoided with relief of the dysmenorrhea.

The case described here is an autopsy of a young woman who committed suicide in which she was incidentally diagnosed with OHVIRA syndrome. This report suggests that OHVIRA syndrome can become a cause of suicide attempt and that intervention for dysmenorrhea may possibly prevent future suicide attempts in adolescent women.

## Abbreviations

OHVIRA syndrome: obstructed hemi-vagina and ipsilateral renal anomaly syndrome

## Declarations

### Availability of data and material

The datasets used and/or analyzed during the current study are available from the corresponding author on reasonable request.

## References

[1] Health and Welfare Statistics Association. Vol. 67, Journal of Health and Welfare Statistics. Health, Labour and Welfare Statistics Association; 2020.

[2] Yoshimasu K, Kiyohara C, Miyashita K, Hygine TSRG of the JS for. Suicidal risk factors and completed suicide: meta-analyses based on psychological autopsy studies. Environ Health Prev Med. 2008;13:243–56. doi: 10.1007/s12199-008-0037-x. 19568911 PMC2698248

[3] Nakao M, Takeuchi T, Yoshimasu K. A proposed approach to suicide prevention in Japan: the use of self-perceived symptoms as indicators of depression and suicidal ideation. Environ Health Prev Med. 2008;13:313–21. doi: 10.1007/s12199-008-0048-7. 19568891 PMC2698228

[4] Chen CX, Shieh DNSc C, Draucker CB, Carpenter JS. Reasons women do not seek health care for dysmenorrhea. J Clin Nurs. 2018;27:e301–8. doi: 10.1111/jocn.13946. 28681499 PMC5746430

[5] Gagua T, Tkeshelashvili B, Gagua D, Mchedlishvili N. Assessment of Anxiety and Depression in Adolescents with Primary Dysmenorrhea: A Case-Control Study. J Pediatr Adolesc Gynecol. 2013 Dec;26(6):350–4. doi: 10.1016/j.jpag.2013.06.018. 24075089

[6] Balik G, Üstüner I, Kağitci M, Şahin FK. Is There a Relationship between Mood Disorders and Dysmenorrhea? J Pediatr Adolesc Gynecol. 2014 Dec 1;27(6):371–4. doi: 10.1016/j.jpag.2014.01.108. 25256879

[7] Selvakumar, Srinivasa Ragaban N, Baskar D, Revathy S. Female suicide death - A multidimensional retrospective analysis. Indian J Forensic Med Toxicol. 2010;4(1):47–52.

[8] Srivastava MK, Sahoo RN, Ghotekar L, Dutta S, Danabalan M, Dutta T, . Risk Factors Associated with Attempted Suicide: A Case Control Study. Indian J Psychiatry. 2004;46(1):3–38. 21206774 PMC2912675

[9] Burgis J. Obstructive Müllerian anomalies’: Case report, diagnosis, and management. Am J Obstet Gynecol. 2001;185(2):338–44. doi: 10.1067/mob.2001.116738. 11518888

[10] Nigam A, Raghunandan C, Yadav R, Tomer S, Anand R. OHVIRA syndrome: rare cause of chronic vaginal discharge in an unmarried female. Congenit Anom. 2011;51(3):153–5. doi: 10.1111/j.1741-4520.2010.00293.x. 20726998

